# Chats about New Books

**Published:** 1887-07-16

**Authors:** 


### CHATS ABOUT NEW BOOKS.

[Books for Review should be sent to the Editor, The Lodge, Porchester Square, W.]

EVERY now and then a new book is published which sets the tongues of the whole reading world wagging, and becomes the sensation of the hour. Usually such books are issued in the forbidding and expensive form of three-volume novels, or two-volume quartos, and it is not until the first edge of excitement as to the book and its author has worn off that there comes a chance for average book-lovers to get access to their pages. Meanwhile anyone who can give a fairly good *resumé* of the story, or describe the work in an attractive manner, is listened to with attention by his own particular coterie of acquaintances. We propose from time to time ourselves to assume the *rôle* of story-teller, and to give to a wider audience than that of the fireside our impressions of some of the more interesting books of the day as they are issued from the press. We shall not attempt to review such books in the ordinary sense of the word. Their grammar, their inherent probability, their plagiarism, we leave to other pens to deal with. If a story successfully beguiles hours of leisure or of weary waiting, it has fulfilled one of the most important of its functions; and it will be sufficient for us if we can so far catch its spirit as to make our chat about the book interesting also. We start with one of the most remarkable books of recent times, by a comparatively new and still youthful writer :—

SHE: a History of Adventure. By H. RIDER HAGGARD (Longmans & Co.). Mr. Haggard has found a new and striking *locale* for his story in the interior of Africa. The mysterious person from whom the novel takes its title is the Queen or head of a race of people called the Amahagger, and she is supposed to have learned the secret of perpetual youth by passing through the sacred fire in the centre of the earth many centuries ago. In the time of the Pharaohs she killed one Kallikrates, whose wife fled northwards and thence to Greece, and is assumed to have inscribed on a potsherd the facts of her husband's death, with an appeal to her infant son to avenge it. The potsherd, with various later inscriptions, is handed down from generation to generation, and at length comes into the hands of a young modern Oxonian, the lineal descendant of Kallikrates, who determines to solve the mystery of the "sherd of Amenartas." He goes to Africa, accompanied by a middle-aged friend and a body-servant. Their remarkable adventures before arriving at their destination, and the still more remarkable events which follow their introduction to the presence of "She," form the groundwork of the story.

Leo Vincey, the hero, falls under the influence of the beautiful and all-powerful She; and at her suggestion goes through thrilling and blood-curdling adventures in order to be endowed, like her, with everlasting youth. To encourage him, she goes through the fire once more, with the unexpected result that she shrivels up immediately and becomes a hideous mummy. The horrified spectators find their way back to the upper air after unparalleled difficulties, and seek refuge from the outside world in Thibet. This is all the plot; the rest is mere incident. But it is incident of a very entrancing kind. It is difficult, having once taken up the book, to put it down again until it is finished, and surely this is the very highest praise that could be given to a book of the kind. One expects to find a good many of the adventures of a high-flown and unusual character; but some border on the impossibly ridiculous. While the glamour of the tale is upon the reader, however, this defect is not much noticed. Mr. Haggard has many faults of style; but he rises on occasion to eloquence. His description of scenery is indeed one of his strongest points. Almost the first chapter of the story, when the necessary preliminary explanations have been given, contains an exceedingly powerful description of a storm and a shipwreck. The concluding words of this chapter we are tempted to quote as a specimen of Mr. Haggard's style :—

"The moon went slowly down in chastened loveliness: she departed like some sweet bride into her chamber, and long veil-like shadows crept up the sky, through which the stars peeped shyly out. Soon, however, they too began to pale before a splendour in the east, and then the quivering footsteps of the dawn came rushing across the new-born blue, and shook the planets from their places. From the east to the west sped the angels of the Dawn, from sea to sea, from mountain top to mountain top, scattering light with both their hands. On they sped out of the darkness, perfect, glorious, like spirits of the dead breaking from the tomb; on, over the quiet sea, over the low coastline, and the swamps beyond, and the mountains beyond them; over those who slept in peace, and those who woke in sorrow; over the evil and the good; over the living and the dead; over the wide world of all that breathes or has breathed thereon."

There are other vivid word-pictures, such as the feast in the cave, which would deserve reproduction if we had space to give them. For this full enjoyment, however, they must be read in the original, and they constitute the chief charm of the story. It is indeed surprising how little one can find to say about the general outline of a book which has had so many spellbound readers, and a success altogether phenomenal.

